# A Comparison of Competences for Healthcare Professions in Europe

**DOI:** 10.3390/pharmacy5010008

**Published:** 2017-02-21

**Authors:** Antonio Sánchez-Pozo

**Affiliations:** Department of Biochemistry, Faculty of Pharmacy, University of Granada, Campus Cartuja s/n, Granada 18071, Spain; sanchezpster@gmail.com; Tel.: +34-958-243-842

**Keywords:** competences, education, pharmacy, healthcare professions

## Abstract

In Europe and elsewhere, there is increasing interest in competence-based education (CBE) and training for professional practice in healthcare. This review presents competences for pharmacy practice in Europe and compares them with those for medicine and dentistry. Comparisons amongst competence frameworks were made by matching the European Directive for Professional Qualifications in sectoral professions such as healthcare (EU directive) with the frameworks of competences elaborated by European consortia in pharmacy (PHAR-QA), medicine (MEDINE), and dentistry (ADEE). The results show that the recommendations of the EU directive for all three professions are similar. There is also widespread similarity in the formulation of competences for all healthcare professions. Furthermore, for medicine and pharmacy, the rankings by practitioners of the vast majority of competences are similar. These results lay the foundations for the design of more interdisciplinary educational programs for healthcare professionals, and for the development of team-based care.

## 1. Introduction

In Europe, and elsewhere in the world, there is an increasing shift from content-based to competence-based education (CBE) and practice. In healthcare sciences, this process started in medicine [1] and is now developing in pharmacy. This shift can bring many advantages. Competences for practice are better understood by the society at large, and thus provide a clearer public statement of the role of the healthcare practitioner. Competences are useful in the mutual recognition of qualifications amongst institutions and government bodies, especially at an international level as amongst European member states. CBE promotes greater comparability and compatibility in educational programs, thus facilitating student and practitioner mobility. The CBE approach also stimulates the development of advanced practice. In European pharmacy, CBE is at present limited; student [2] and practitioner mobility is low [3], and advanced practice, although developing, is still not recognized by the EU [4].

Competence frameworks for pharmacy education have emerged during the last years both at national, European, and worldwide levels. These have been promoted by professional chambers and associations, and academia [5,6,7,8,9,10,11,12,13,14,15]. European frameworks have been proposed for other healthcare sciences such as medicine (MEDINE: Medical education in Europe) [16] and dentistry (ADEE: Association for Dental Education in Europe) [17].

In this paper, we compared the CBE framework for EU pharmacies developed by the PHAR-QA (Quality assurance in European pharmacy education and training) [13] consortium (a follow-up to the PHARMINE (Pharmacy education in Europe) [14] project) with those for medicine (MEDINE [16]) and dentistry (ADEE [17]).

The comparison was carried out in three parts:

1. The recommendations for the minimum requirements of the EU directive [4].
2. The formulations of the academic proposals for CBE in healthcare sciences.
3. The perception by practitioners of the framework proposals for pharmacy and medicine. (This step has not to our knowledge been undertaken in dentistry).

EU Directives of the European Parliament and of the Council on the recognition of professional qualifications have consolidated a system of mutual recognition. It provides for automatic recognition for a limited number of professions based on harmonized minimum training requirements (sectoral professions), a general system for the recognition of evidence of training and automatic recognition of professional experience. The directives have also established a new system of the free provision of services.

Evaluation of the perception of practitioners is an essential step in building a framework. To do this, practitioners rank the competences proposed according to their own development needs, after reflection on the competences required for their particular professional practice. Faculties and other academic institutions have collaborated in the establishment of a framework of competences based on the scientific advances and new methodologies in education. Examples of this collaboration include the PHARMINE and MEDINE. However, the academic knowledge of the problems have to be tested in the working places. This dual approach—an academic proposal followed by ranking by practitioners—is an integral part of the production of a viable framework.

## 2. Results and Discussion

### 2.1. The Recommendations for Minimum Requirements of the European Directive

The 2013 EU directive on the recognition of professional qualifications [4], an amendment of the 2005 EU directive [18], deals mainly with structural management issues, such as length of degree course and the attributes of training, rather than competences. It does, however, set out a series of minimum requirements for the healthcare sciences (Table 1).

As shown in Table 1, the requirements of the EU directive for the sectoral professions of pharmacy, medicine (general practice), and dentistry have many things in common. Education and training for all three types of practitioner require basic science, human physiopathology, and clinical experience.

Only the requirements for medicine and dentistry, however, emphasize clinical disciplines in which the professional is in direct contact with healthy or sick individuals. However, there has been an evolution in pharmacy from the EU directive in its 2005 version [18] to its 2013 version [4] (Table 2), with the installation of a more “clinical” role for pharmacists as far as patient centered care and public health is concerned. Others professions such as nurses and midwives have also had changes in their requirements, whereas medicine and dentistry remain unchanged.

It should be noted that the elements given in Table 1 and Table 2 are not competences. They describe knowledge or activities. For instance, the requirement “the sciences upon which practice is based” corresponds to the levels (“knows” and “knows how”) of Miller’s triangle [19]; or the “Provision of information and advice on medicinal products” corresponds to the levels (“shows how” and “does”). However, the EU directive still lacks detail on “competences for practice” and this is one of the reasons why the PHAR-QA, MEDINE, and ADEE academic consortia produced their detailed frameworks for pharmacy, medicine, and dentistry, respectively.

### 2.2. The Formulations of the Academic Proposals for CBE in Healthcare Sciences

A comparison was made of the competence frameworks proposed by academia for pharmacy (PHAR-QA), medicine (MEDINE), and dentistry (ADEE). The major competences were divided into domains as shown in Table 3. We grouped the competences in clusters of related competences: first in groups of very close competences that we called major competences, and then in domains of related major competences. For example, the major competence “professional attributes” includes competences such as probity, honesty, commitment to maintaining good practice, concern for quality, critical and self-critical abilities, reflective practice, and empathy, and the domain “professionalism” includes professional attributes, professional work, and ability to apply ethical and legal principles. This grouping facilitates comparisons, as the individual definitions of competences by the three consortia concerned are not always identical, even though they are talking about the same concept.

The following domains are common to all three professions: professionalism, interpersonal competences, communication and social skills, knowledge base, information and information literacy, clinical information gathering, diagnosis and treatment planning, therapy, establishing and maintaining health, and prevention and health.

The major competences included in the domains of Table 3 account for more than 95% of the major competences described in the frameworks. They can thus be considered as representative of the frameworks proposed.

For each domain, peer major competences appear on the same line, whereas non-equivalent major competences appear on different lines (Table 3). There are gaps in the table (perhaps) representing major competences that (one or more) professions consider implicit.

The first three domains relate to personal competences and are very similar in all healthcare professions. A specific attitude and behavior to patients, together with an ethical commitment, are common aspects of these healthcare professions. Communication and social skills are clearly needed for the information and education of patients. As in many other professions, the use of information technology and the ability to solve problems is a common denominator.

The last four domains (4–7) comprise the specific competences related to patient care. Patient care requires (1) clinical judgment based on competences for gathering information included in the domain “Clinical information gathering”; (2) assessment and treatment planning, included in the domain “Diagnosis and Treatment planning”; and (3) monitoring the results, included in the domain “Therapy, establishing and maintaining health.” These latter domains are present in all three healthcare professions. We suggest that the decisions about the need for a drug, the selection, dosage, the adverse effects, etc., typically performed by the pharmacist, follow the same principles as other clinical disciplines and thus require the same competences. This is reflected in the increasing role of pharmacists in patient care as recognized by the EU (see above).

### 2.3. The Perception by the Practitioners of the Framework Proposals for Pharmacy and Medicine

In the PHAR-QA [14] and MEDINE [16] projects the competences were ranked by practitioners in each profession.

**Figure 1:**
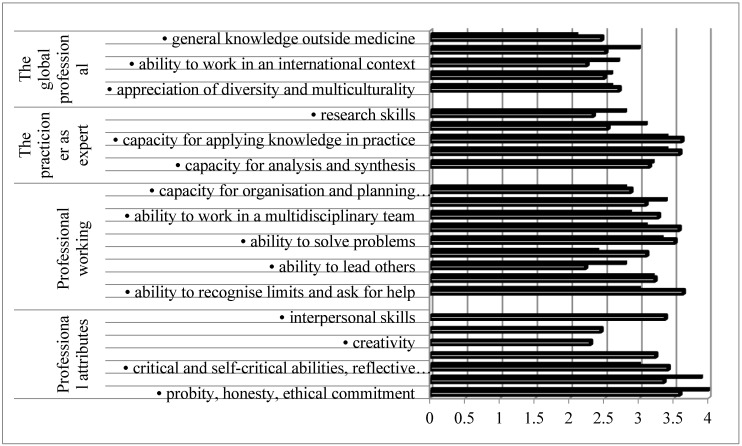
Ranking of personal competences by pharmacy (full columns) and medicine (open columns) practitioners. Ranking was on a 4-point scale (1 = least and 4 = most important). Pharmacy data are from PHAR-QA [19,20,21,22], and for medicine MEDINE [16].

Figure 1 shows that all competences were considered “necessary” (rank > 2/4), although with a considerable degree of variability. Globally, ranking scores for pharmacists and general practitioners were similar, although there were some differences. Knowledge of a second language and research skills were ranked higher by pharmacists; competences such as the ability to work autonomously and to recognize limits were ranked higher by general practitioners.

**Figure 2:**
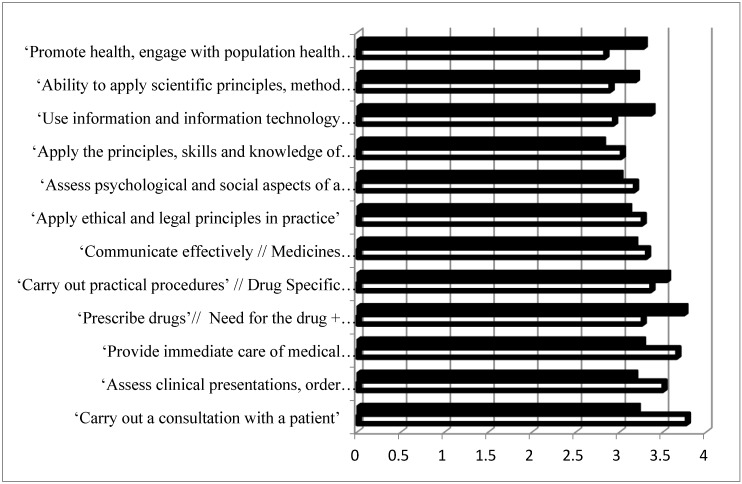
Ranking of patient care competences by pharmacy (full columns) and medicine (open columns) practitioners. Ranking was on a 4-point scale (1 = least and 4 = most important). Pharmacy data are from PHAR-QA [19,20,21,22], and for medicine MEDINE [16].

All patient care competences were considered “necessary” (rank >2/4) (Figure 2). The spread for patient care competences (2.9–3.8) was higher than for personal competences (2–3.8), suggesting that all practitioners rank patient care competences as more important. Rankings were similar for pharmacy and medicine with the global rank being lower for pharmacy than for medicine (delta = −0.5).

## 3. Conclusions

The results show that there is much similarity in competences for practice amongst all healthcare professions. This is seen in the recommendations for practice in the EU directive. It is also seen in the formulation of the competences by the different EU academic consortia that have proposed competence frameworks for pharmacy (PHAR-QA, medicine (MEDINE) and dentistry (ADEE)). Finally, it is seen in the perception of pharmaceutical and medical practitioners through their relative ranking of the proposals for competences.

The identification of a large number of competences that are similar in healthcare professions opens up the possibilities of a new design in educational programs with the installation of CBE, of more interaction in the different healthcare disciplines regarding education and practice, and, globally, of programs that are more adequate to an era of team-based healthcare.

## Figures and Tables

**Table 1:** The minimum requirements for healthcare professions as given in the 2005 EU directive [4].

Requirement	Pharmacy	Medicine	Dentistry
The sciences upon which practice is based	X	X	X
The scientific methods including the principles of measurement	X	X	X
Evaluation of scientific data	X	X	X
Structure, function and behavior of healthy and sick persons	X	X	X
Traineeship in a community or hospital setting	X	X	X
Clinical disciplines and practices		X	X

**Table 2:** Description of the roles of pharmacist given in the 2005 and 2013 EU directives. Differences in EU directives concerning patient care and public health issues are given in bold.

EU Directive 2005 [18]	EU Directive 2013 [4]
Preparation of the pharmaceutical form of medicinal products; manufacture and testing of medicinal products; testing of medicinal products in a laboratory for the testing of medicinal products; storage, preservation and distribution of medicinal products at the wholesale stage	Same as 2005
Preparation, testing, storage and supply of medicinal products in pharmacies open to the public	Ordering, manufacture, testing, storage and dispensing of safe, high quality medicinal products in public pharmacies
Preparation, testing, storage and dispensing of medicinal products in hospitals	Same as 2005
Provision of information and advice on medicinal products	Medication management and provision of information and advice about medicinal products and **general health information**
	**Provision of advice and support to patients in connection with the use of non-prescription medicines and self-medication**
	**Contributions to public health and information campaigns**

**Table 3:** Domains and major competences in frameworks of competences for the pharmacist (PHAR-QA), general medical practitioner (MEDINE) and dentist (ADEE).

Domain	Major Competences		
	PHAR-QA	MEDINE	ADEE
1. Professionalism	Personal competences: values.	Professional attributes	Professional attitude and behavior
	Personal competences: learning and knowledge.	Professional working	
	Personal competences: values.	Apply ethical and legal principles	Ethics and jurisprudence
2. Interpersonal, communication and social skills	Personal competences: communication and organizational skills.	Communicate effectively in a medical context	Communication
3. Knowledge base, information and information literacy	Personal competences: learning and knowledge.	Apply the principles, skills and knowledge of evidence-based medicine	Application of basic biological, medical, technical and clinical sciences
	Personal competences: learning and knowledge.	Use information and information technology effectively in a medical context	Acquiring and using information
4. Clinical information gathering	Patient care competences: patient consultation and assessment.	Carry out a consultation with a patient	Obtaining and recording a complete history of the patient’s medical, oral and dental state
		Assess psychological and social aspects of a patient’s illness’	
5. Diagnosis and Treatment planning	Patient care competences: need for drug treatment.	Assess clinical presentations, order investigations, make differential diagnoses and negotiate a management plan	Decision-making, clinical reasoning and judgment
	Patient care competences: drug interactions.	Provide immediate care of medical emergencies, including First Aid and resuscitation’	
	Patient care competences: drug dose and formulation.		
	Patient care competences: provision of information and service.		
6. Therapy, establishing and maintaining health	Patient care competences: monitoring of drug therapy.	Carry out practical procedures	Establishing and maintaining oral health
		Prescribe drugs	
7. Prevention and health promotion	Patient care competences: patient education.	Promote health, engage with population health issues and work effectively in a health care system	Improving oral health of individuals, families and groups in the community
